# Correction: Metal-free [2+2+1] cycloaddition polymerization of alkynes, nitriles, and oxygen atoms to functional polyoxazoles

**DOI:** 10.1039/d0ra90139c

**Published:** 2021-01-11

**Authors:** Lichao Dong, Tian Lan, Yin Liang, Shifeng Guo, Hao Zhang

**Affiliations:** The 306th Institute of China Aerospace Science & Industry Corp. Beijing 100074 China 610557405@qq.com

## Abstract

Correction for ‘Metal-free [2+2+1] cycloaddition polymerization of alkynes, nitriles, and oxygen atoms to functional polyoxazoles’ by Lichao Dong *et al.*, *RSC Adv.*, 2020, **10**, 24368–24373, DOI: 10.1039/D0RA04249H.

The authors regret that the funding information given in the original manuscript was incorrectly shown. The authors declare that there was no financial support for this research.

The Royal Society of Chemistry apologises for these errors and any consequent inconvenience to authors and readers.

## Supplementary Material

